# A high FIB-4 score may be an early sign of severe liver involvement in patients with primary antibody deficiencies

**DOI:** 10.55730/1300-0144.6219

**Published:** 2026-03-13

**Authors:** Hacı Uğur MUŞABAK, Tuba ERDOĞAN, Emre ÖZBEK, Dudu Merve DURAK ANŞİN, Zumrud MEHTİ

**Affiliations:** 1 Division of Immunology and Allergy, Baskent University, Faculty of Medicine, Ankara, Turkiye; 2 Department of Immunology-Allergy, Etlik City Hospital, Ankara, Turkiye

**Keywords:** Common variable immunodeficiency, fibrosis-4 index, liver damage

## Abstract

**Background/aim:**

Liver damage is a significant complication that increases mortality and morbidity in primary immunodeficiencies (PIDs). Although biopsy is the gold standard, the fibrosis-4 (FIB-4) scoring system is a noninvasive method for diagnosing liver fibrosis. In this study, we investigated the value of the FIB-4 score as an early indicator of liver damage in patients with primary antibody deficiencies (PADs).

**Materials and methods:**

Of 52 patients, 40 had common variable immunodeficiency (CVID), 12 had immunoglobulin G subclass deficiency (IgG-SGD), and they were enrolled in the study. Patients were divided into hematology, rheumatology + dermatology, gastroenterology/hepatology, and respiratory diseases groups based on their clinical phenotypes. FIB-4 score calculation and transient elastography were used to detect liver complications.

**Results:**

FIB-4 scores were significantly higher in CVID patients than in those with SGD (p = 0.003), and were also elevated in PAD patients with hematology and gastroenterology/hepatology phenotypes compared with rheumatology + dermatology and respiratory phenotypes (p < 0.001). In the gastroenterology/hepatology group patients, FIB-4 scores showed a strong positive correlation with elastography values (*r* = 0.825, p < 0.001). FIB-4 and elastography showed moderate to good agreement (κ = 0.41–0.60) for detecting significant fibrosis, and FIB-4 was also a valuable indicator in high-risk patients (≥3.25).

**Conclusions:**

This study is the first to show that FIB-4 may serve as a practical and noninvasive indicator of liver damage in PADs.

## 1. Introduction

Primary antibody deficiencies (PADs) account for more than half of primary immunodeficiencies, and some have identifiable genetic defects [1]. Common variable immunodeficiency (CVID), IgG subclass deficiencies (IgG-SGDs), specific antibody deficiencies (SADs), and selective IgA deficiency (sIgA-D) are the most commonly diagnosed PADs. These diseases are characterized by hypogammaglobulinemia and increased susceptibility to infections, including upper respiratory tract infections, pneumonia, and gastrointestinal infections, predominantly bacterial. It should be noted that patients can be seen in many different clinics based on their chief complaints [2]. Disturbances in immunoregulatory processes can lead to the emergence of hematological, gastroenterological, rheumatological, dermatological, and endocrinological autoimmune diseases and malignancies [1, 2].

Common variable immunodeficiency is one of the leading causes of PADs and is frequently symptomatic in adults [3]. Liver damage in patients with CVID has been associated with increased mortality and morbidity. When this complication develops, the clinical spectrum ranges from asymptomatic elevations in common liver enzymes to portal hypertension and primary biliary cirrhosis [4,5]. Histopathological features of liver disease in CVID include mild nonspecific portal and lobular hepatitis, absence of plasma cells, and nodular regenerative hyperplasia (NRH) [6]. Although histopathological assessment of liver biopsies is the gold standard for evaluating liver disease, this method is not always feasible or easy [7]. Therefore, noninvasive methods such as serological panels and radiological imaging are at the forefront of the follow-up of liver diseases and related complications.

Transient elastography is a new noninvasive imaging technique that measures the stiffness of internal organs [7]. Liver elastography, using ultrasound or magnetic resonance imaging, enables noninvasive stratification and monitoring of fibrosis in liver disease. Several studies in the literature have evaluated liver damage using elastography in cohorts with CVID. Globig et al. showed that high liver stiffness (>6.5 kPa) measured by transient elastography was associated with progression to portal hypertension in patients with CVID, while DiGiacomo et al. established a cutoff value of 6.2 kPa for NRH in CVID using the same method [8, 9]. Crescenzi et al found increased transient elastography values correlated with polyclonal lymphoproliferation and enteropathy in up to one-third of patients in their CVID cohort [10]. However, when the literature is carefully examined, it is clear that data on elastography for the follow-up of liver damage in PADs are limited, and there is no comprehensive research on this subject.

In 2006, Sterling et al proposed a serological panel, fibrosis-4 (FIB-4) score, calculated based on a formula including age, platelet, alanine aminotransferase (ALT), and aspartate aminotransferase (AST) to predict the severity of liver fibrosis for human immunodeficiency virus (HIV) and hepatitis C virus (HCV) coinfected patients [11]. According to our literature review, although the FIB-4 score has been extensively investigated for nonalcoholic fatty liver disease (NAFLD) and nonalcoholic steatohepatitis (NASH), no study has yet been conducted using this score as a noninvasive method to demonstrate liver damage in PAD [12–14]. Therefore, in this study, our primary outcome was to evaluate the diagnostic and prognostic utility of the FIB-4 score in detecting liver damage among PAD patients. As a secondary outcome, we aimed to investigate the potential correlations between this score and immunological parameters in distinct clinical phenotypes of the disease.

## 2. Materials and methods

### 2.1. Patients

A total of 52 adult patients (13 men, 39 women) with PAD who were regularly followed up in our clinic between January 2017 and July 2025 were enrolled in this study (Table 1). In the first stage of the study, patients were divided into two groups: 40 patients diagnosed with CVID and 12 patients diagnosed with IgG-SGD according to the European Society for Immunodeficiencies (ESID) diagnostic criteria [15]. Of the patients with IgG-SGD, five had IgG2 + IgG4 deficiency, two had IgG2 + IgG3 deficiency, and two had IgG3 deficiency. One patient each had IgG2 + IgG3 + IgG4 deficiency, IgG3 + IgG4 deficiency and IgG4 deficiency. In the second stage of the study, all patients were phenotypically divided into four groups according to their predominant clinical signs and symptoms, without distinction between CVID and SGD groups: a) hematology, b) rheumatology + dermatology, c) gastroenterology/hepatology, and d) respiratory diseases (Table 1). This organ-system-based classification was specifically adopted to evaluate the clinical utility and performance of the FIB-4 score across diverse clinical manifestations, with particular emphasis on the gastroenterology/hepatology phenotype. While PAD cohorts are often categorized by the presence or absence of noninfectious complications, our approach allowed for a more granular assessment of liver-specific markers within a multidisciplinary clinical framework.

The distribution of patients’ diagnoses other than PAD by clinical phenotype is as follows. In the hematology group, two of the seven patients had ITP, one had immune neutropenia, 2 had non-Hodgkin lymphoma, and two had B-cell acute lymphoblastic leukemia. These diagnoses were made before the PAD diagnosis, and all patients with hematologic malignancies were in remission. In the rheumatology + dermatology group, three of the 10 patients had rheumatoid arthritis, two had systemic lupus erythematosus, two had spondyloarthritis, two had pemphigus vulgaris, and one had bullous pemphigoid. Three patients had dermatological signs and symptoms and were added to the rheumatology group. In the gastroenterology/hepatology group, four of the 14 patients had ulcerative colitis, three had Crohn’s disease, two had ulcerative colitis + Crohn’s disease, three had NRH, one had type 1 autoimmune hepatitis, and one had Celiac disease. One of these 14 patients had hepatitis B virus, and one had hepatitis C virus carriage. Of the remaining 12 patients, two had grade 1 steatosis, and three had grade 2 steatosis. In this study, the term “liver damage” encompasses a broad spectrum of hepatic pathologies, ranging from transient biochemical elevations to advanced architectural changes. A systematic classification of these manifestations is provided in Table 2. Of the 45 patients in groups other than the hematology clinical phenotype, 32 were diagnosed with comorbid diseases at the time of PAD diagnosis. Twelve of these patients were receiving maintenance dose immunosuppressive therapy due to their comorbidities. All 21 patients in the respiratory disease group had a history of frequent upper respiratory tract infections, and 13 reported being treated at another center for pneumonia.

Patients with active malignancies or those with secondary immunodeficiency clearly attributable to other underlying diseases, protein-losing diseases (nephropathy, enteropathy), or HIV infection] were excluded. Patients with a history of hematologic malignancies (e.g., lymphoma or leukemia) who were in complete remission and had a confirmed underlying diagnosis of PAD were included.

Medical history, demographic data, and laboratory records of patients were examined retrospectively. In patients receiving maintenance dose immunosuppressive therapy at the time of hospital admission, the diagnosis of PAD was made by careful review of clinical data before initiating these treatments. No death was observed during the follow-up period. All patients received regular intravenous immunoglobulin (IVIG) therapy calculated based on body weight. Antibiotic prophylaxis with trimethoprim/sulfamethoxazole and empiric antimicrobial therapy were prescribed as needed.

This study was approved by the Başkent University Institutional Review Board and Ethics Committee (Project no: KA23/382). Written informed consent was received from all the participants. The study was conducted in accordance with the Declaration of Helsinki.

### 2.2. Hematological parameters

Complete blood counts were analyzed by an automated hematology analyzer (Cell-Dyn Ruby, Abbot Diagnostics, Santa Clara, CA, USA). The test results were evaluated according to the manufacturer’s reference range.

### 2.3. Immunological parameters

While IgG, IgM, and IgA levels were measured with the turbidimetric method using Clinical Chemistry Analyzer (Architect C8000, Abbot Diagnostics, Santa Clara, CA, USA), total IgE levels were measured with the enzyme immunoassay (EIA) method using IMMULITE 2000 chemiluminescent EIA (CLEIA) system (Siemens Healthcare Diagnostics, Deerfield, IL, USA). Reference ranges for immunoglobulin measurements are: IgG: 7.1–16 g/L; IgM: 0.3–2.9 g/L; IgA: 0.7–5.2 g/L; and total IgE < 87 IU/mL.

Flow Cytometry (Becton, Dickinson and Company, BD Biosciences, FACSCanto II system) was used to analyze lymphocyte subsets. The same company provided fluorescence-labelled monoclonal antibodies and other reagents appropriate for the device. Accordingly, the percentages of peripheral blood CD3^+^ T cells, CD4^+^ T cells, CD8^+^ T cells, CD19^+^ B cells, CD45^+^ lymphocytes, and CD16 + CD56 + CD3^−^ natural killer cells were identified by BD Multitest 6-Colour TBNK reagent, [Becton, Dickinson (BD) and Company, BD Biosciences, San Jose, CA, USA].

After the patients’ blood samples were collected into EDTA-containing tubes, lymphocyte subsets were studied on the same day. For this purpose, 50 mL of reagents were added to 100 mL of blood samples, and the mixture was slowly vortexed. The mixture was incubated for 20 min at 20–25 °C. Then, red blood cells were lysed by FACS lysing solution (BD Company) and removed by washing with phosphate-buffered saline (PBS). At the end of this process, the cells were passed through the laser of the flow cytometer and analyzed by the software (BD FACSCanto Clinical Software).

Serum alanine aminotransferase (ALT), aspartate aminotransferase (AST) level, albumin, total protein, platelet count, erythrocyte sedimentation rate (ESR), and C-reactive protein (CRP) level were sampled at the first admission, and FIB-4 scores were calculated by the formula proposed by Sterling et al [11].


$$\text{FIB-}4=[\text{age }(\text{years})\times \text{AST }(\text{IU}/\text{L})]/[\text{platelet count }(10^{9}/\text{L})\times [\text{ALT }(\text{IU}/\text{L})]1/2].$$

In the study by Sterling et al., 832 patients, categorized according to the Ishak histopathological scoring system, were reevaluated using the FIB-4 scoring system. Accordingly, a cutoff of <1.45 had a negative predictive value of 90% with a sensitivity of 70%, while a cutoff of >3.25 had a positive predictive value of 65% with a specificity of 97%.

The FibroScan device, using the transient elastography technique, was used to measure the degree of liver fibrosis in gastroenterology/hepatology patients with clinical indications such as hepatomegaly, persistently elevated liver enzymes, or clinical suspicion of liver damage [16]. Accordingly, the previously suggested cutoff value for this technique (>9.6) was used in our study to indicate severe liver fibrosis. This approach enabled targeted validation of noninvasive fibrosis scores (FIB-4) against elastography in high-risk patients within the cohort.

## 3. Statistical analysis

Statistical analysis was performed using IBM SPSS Statistics for Windows v.21.0 (IBM Corp., Armonk, NY, USA). The one-sample Kolmogorov–Smirnov test was used to determine the normality of the distribution of numerical parametric data. Multiple comparisons of numerical data were performed using the one-way ANOVA or the Kruskal–Wallis test, whichever was appropriate. Then, the differences between the two groups were evaluated using the t-test or the Mann–Whitney *U* test. Chi-square test was also used for comparisons of categorical data. In addition, the relationships between all variables were investigated using Spearman’s rank test. A simple linear regression model was also used to estimate the relationship between the dependent and independent variables. Cohen’s kappa coefficient was calculated to measure the degree of agreement between two categorical results. All directional p-values were two-tailed, and the level of statistical significance was set as p < 0.05.

## 4. Results

The average age of all patients was 46.1 ± 16.7 years, and the IVIG dose they received every 3 wk was 38.1 ± 5.6 g. While there was a statistical difference between the mean ages of all patients according to gender (female: 50.2 ± 16.3; male: 33.7 ± 11.4; p = 0.001), there was no statistical difference between IVIG dose given every 3 wk (38,1 ± 5.6 g) and distribution of patients with CVID and IgG-SGD (75% vs. 25%) (p>0.05 for both comparisons).

A statistical difference in the distribution of clinical phenotypes was detected between patients with CVID and IgG-SGD (p < 0.001). Accordingly, the distribution of clinical phenotypes in the CVID patient group, in order of frequency, is 40% for respiratory diseases, 35% for gastroenterology/hepatology, 17.5% for hematology, and 7.5% for rheumatology + dermatology. In the patient group with SGD, the rates of rheumatology + dermatology were 58.3%, and those of respiratory diseases were 41.7%. No patients with other clinical phenotypes were included in this group.

### 4.1. Comparisons of patients with CVID and IgG-SGD

**Figure 1:**
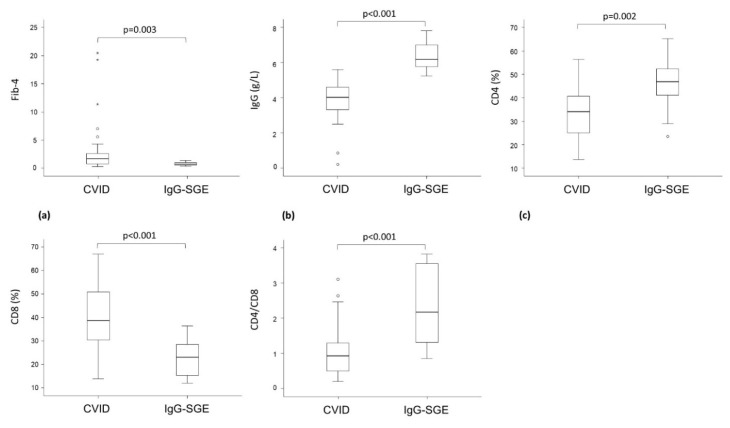
Comparison of patients with CVID and IgG subgroup deficiency in terms of FIB-4 index and immunological parameters. The boxes show the ranges of the 1st and 3rd quartiles and extreme values, with the thick horizontal bars representing median values. The differences between the two independent groups were evaluated using the Mann–Whitney *U* test. The p-values are shown above the boxes when the significance level is <0.05 in comparisons between the study groups.

There was no statistical difference between these patient groups in terms of age and gender (Table 1). However, IVIG doses were significantly higher in patients with CVID (Table 1). Additionally, no difference was observed between patients with CVID and IgG-SGD in ESR and CRP levels, which are indicators of clinical activity. However, in the complete blood count, hemoglobin, leukocyte, neutrophil, lymphocyte, and platelet counts were statistically significantly lower in the first group than those of the second group. In routine biochemistry tests, while the total protein level was significantly lower in the CVID group compared with the IgG-SGD group, there was no significant difference between the other measured parameters (Table 1). The FIB-4 score was found to be statistically significantly higher in the CVID patient group than in the SGD patient group (Table 1, Figure 1a).

Differences were also detected between the immunological parameters measured in patients with CVID and IgG-SGD (Table 1, Figure 1). The IgG level in the CVID group was significantly lower than the SGD group (As shown in Figure 1b). Similarly, the percentage of CD4^+^ T cells and the CD4/CD8 ratio were significantly lower in the CVID patient group (as shown in Figure 1c, e). While the percentage of CD8^+^ T cells was significantly higher in patients with CVID (as shown in Figure 1d), there was no difference between the groups in terms of CD3^+^ T cells, CD19^+^ B cells, and CD3^−^CD16^+^56^+^ NK cells. IgA, IgM, and IgE levels were compared between groups by the percentage of patients with measurable immunoglobulin isotype levels (Table 1). Accordingly, while the percentages of measurable IgA and IgM were significantly lower in the CVID patient group, there was no significant difference in IgE between the groups.

### 4.2. Correlations between FIB-4 scores and other measured parameters in patients with CVID and IgG-SGD

In the CVID patient group, there was a positive correlation between the FIB-4 score and acute-phase indicators (ESR with *r* = 0.348, p = 0.028; CRP with *r* = 0.385, p = 0.014). The effects of ESR and CRP levels on the FIB-4 score were statistically significant by simple linear regression analysis (p = 0.028, 0.014, respectively). A negative correlation was detected between the FIB-4 score and some complete blood values and biochemical tests (with hemoglobin: *r* = −0.372, p = 0.018; with leukocyte count: *r* = −0.419, p = 0.007; with neutrophil count: *r* = −0.410, p = 0.009; with albumin: *r* = −0.348, p = 0.028; with total protein: *r* = −0.482, p = 0.002). The effects of hemoglobin, leukocyte, and neutrophil counts, albumin, and total protein levels on FIB-4 score were found to be statistically significant by simple linear regression analysis (p = 0.018, 0.007, 0.009, 0.028, 0.002, respectively). No correlation was found between FIB-4 scores and immunological parameters in CVID patients. In patients with IgG-SGD, the FIB-4 score did not correlate with any of the measured parameters.

Other statistical relationships were also found between biochemical tests and immunological tests in the CVID patient group. However, these data were not given here because they were beyond the scope of the study.

### 4.3. Comparisons of patients according to clinical phenotypes

When patients were divided by clinical phenotype, regardless of CVID or IgG-SGD status, they were grouped into four groups with different frequencies (Table 1). In this case, significant differences in age and gender were observed between the clinical phenotypes. While age was significantly higher in the hematology group than in the gastroenterology/hepatology and respiratory diseases groups, it was not different from other clinical phenotypes. The age in the rheumatology + dermatology group was significantly higher than in the gastroenterology/hepatology and respiratory diseases groups, while the age in the gastroenterology/hepatology group was significantly higher than that in the respiratory diseases group. Although the gender distribution was similar in the respiratory disease group, female predominance was observed in other clinical phenotypes. No difference was found between the patient groups in IVIG doses.

**Figure 2:**
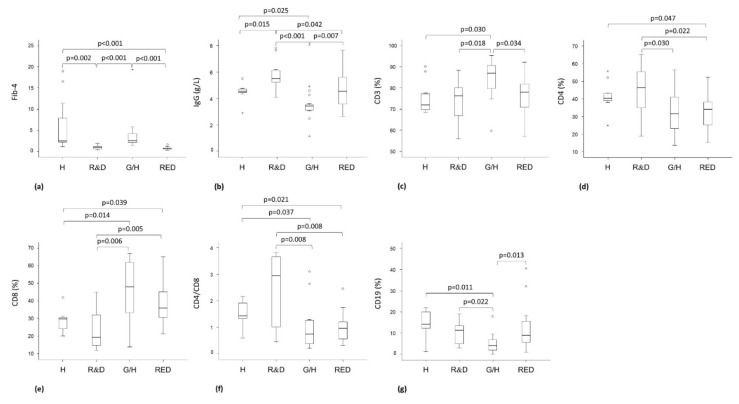
Comparison of patients with primary antibody deficiencies classified into clinical phenotypes in terms of FIB-4 index and immunological parameters. The boxes show the ranges of the 1st and 3rd quartiles and extreme values, with the thick horizontal bars representing median values. The Mann-Whitney U test was used to evaluate differences between the two independent groups. The p-values are shown above the boxes when the significance level is <0.05 in comparisons between the study groups. Abbreviations: H: hematology, R&D: rheumatology and dermatology, G/H: gastroenterology/hepatology, RED: respiratory diseases (chest, ear, nose, and throat).

While no significant differences in ESR and CRP were observed among patients with different clinical phenotypes, notable differences in complete blood counts were observed across these phenotypes (Table 1). In biochemical tests, AST levels were higher in the gastroenterology/hepatology group than in the other groups, except the hematology group. However, the total protein level in the gastroenterology/hepatology group was significantly lower than in the other groups. Significant differences were also observed between clinical phenotype groups in FIB-4 scores. Accordingly, FIB-4 scores did not differ between the gastroenterology/hepatology and hematology groups, but those of both groups were significantly higher than those of the rheumatology + dermatology and respiratory diseases groups (Figure 2a). The FIB-4 score in the rheumatology + dermatology group was not different from that of the respiratory diseases group.

There were also statistically significant differences in immunological parameters between clinical phenotypes (Table 1). While IgG levels were significantly lower in the gastroenterology/hepatology group than in the other groups, they were significantly higher in the rheumatology + dermatology group than in the other groups (Figure 2b). There were differences in the frequency of patients producing detectable levels of IgA, IgM, and total IgE across clinical phenotypes. IgM production in patients in the gastroenterology/hepatology group and IgA production in patients in the hematology group were below detectable limits. The highest frequency of IgA production was observed in the rheumatology + dermatology group, while the highest frequency of IgM production was observed in the hematology group. There was no difference between the groups in total IgE levels.

The percentage of CD3^+^ T cells was significantly higher in the gastroenterology/hepatology group than in the other groups (Figure 2c). The lowest percentage of CD3^+^ T cells was in the hematology group. CD4^+^ T cell percentage was higher in the rheumatology + dermatology group than in the gastroenterology/hepatology and respiratory diseases groups, but not different from the hematology group (Figure 2d). CD4^+^ T cell percentage in the hematology group was also higher than in the respiratory diseases group. The percentages of CD8^+^ T cells in the gastroenterology/hepatology and respiratory diseases groups were higher than those in the other groups, but did not differ between the two groups (Figure 2e). Accordingly, the CD4/CD8 ratios detected in the gastroenterology/hepatology and respiratory diseases groups were lower than those detected in the other groups. (as shown in Figure 2f). The percentage of CD19^+^ B cells was significantly lower in the gastroenterology/hepatology group than in the other groups (Figure 2g). There was no difference between clinical phenotypes in terms of CD3^–^CD16^+^56^+^ cell percentages.

### 4.4. Correlations between FIB-4 scores and measured parameters in patients according to clinical phenotypes

A strong, statistically significant positive correlation was found between FIB-4 score and Elastography values in patients in the gastroenterology/hepatology group (*r* = 0.825, p < 0.001). When simple regression analysis was performed with the FIB-4 score as the independent variable and the elastography result as the dependent variable, the relationship detected by the correlation analysis was confirmed in both its strength and statistical significance (*r* = 0.803, p < 0.001). The FIB-4 index had strong predictive power, explaining approximately 64.5% of the variation in elastography values (R^2^ = 0.645).

Cohen’s kappa coefficient (k) was calculated to determine the degree of agreement between the generally accepted FIB-4 risk categories and fibrosis categories determined by Elastography [17, 18]. For this purpose, FIB-4 and Elastography results were divided into two simpler and clinically meaningful categories: 1) No Significant Fibrosis (Low/Moderate Risk): FIB-4 Threshold <3.25, Elastography Threshold <9.6 kPa, Fibrosis Stage: F0–F2. 2) Significant Fibrosis (High Risk/Advanced Fibrosis): FIB-4 Threshold ^3^ 3.25, Elastography Threshold ^3^ 9.6 kPa, Fibrosis Stage: F3–F4. A contingency table was then created to calculate Cohen’s kappa coefficient (Table 3). According to the formula used to calculate Cohen’s kappa coefficient, this value was found to be 0.588.


k=[P(A)-P(E)]/[1-P(E)]; P(A)=number of agreements,P(E)=number of expected agreements.

The calculated value (k = 0.588) was interpreted according to the table suggested by Landis and Koch [19]. Accordingly, it was concluded that the FIB-4 score and elastography results showed moderate to good agreement (*r* = 0.41–0.60) when categorized by the presence or absence of significant fibrosis.

In hematology group patients, the FIB-4 score showed a negative correlation with both IgG levels (*r* = −0.876, p = 0.010) and CD19^+^ B cell percentage (*r* = −0.768, p = 0.044), and a positive correlation with CRP levels (*r* = 0.821, p = 0.023). The effects of IgG levels, CD19^+^ B cell percentage, and CRP levels on the FIB-4 score were statistically significant by simple linear regression (p = 0.010, 0.044, and 0.023, respectively).

No significant correlation was found between the parameters in patient groups with other clinical phenotypes.

### 4.5. Multivariable analysis of factors associated with FIB-4 scores in the entire PAD cohort

To identify independent predictors of elevated FIB-4 scores across the entire cohort (n = 52), a multivariable regression analysis was performed, incorporating clinically relevant immunological parameters and inflammatory markers (excluding direct components of the FIB-4 formula). Since transient elastography was exclusively performed in the gastroenterology/hepatology subgroup, it was not included in the multivariable model for the entire cohort to maintain statistical power and avoid excessive missing data. However, no significant independent associations were identified in the full cohort, likely reflecting the high degree of clinical and immunological heterogeneity among PAD patients (data not shown).

## 5. Discussion

IgG-SGDs, which constitute an important subgroup of PADs, are diseases with the potential to progress to CVID. When relatives of patients with CVID are screened, other PADs can be detected in addition to IgG-SGDs [1]. When all PADs are considered together, they are recognized as a clinically and immunologically heterogeneous group of diseases. This heterogeneity has negatively impacted disease diagnosis and management, prompting researchers to conduct large-scale studies to characterize the clinical and immunological phenotypes of the disease.

The retrospective study by Chapel et al. is one of the pioneering studies conducted for this purpose [20]. In this multicenter study, data from 334 patients with CVID were evaluated, and patients were grouped according to different clinical phenotypes, including those who did not develop complications and those who developed autoimmunity, polyclonal lymphocytic infiltration, enteropathy, or lymphoid malignancy. In the multicenter EUROClass study, 303 patients with CVID exhibited the clinical phenotypes of autoimmune cytopenia, granulomatous disease, and splenomegaly, and the latter two phenotypes were associated with a severe decrease in switched memory B cells [21]. A more recent study showed that the frequency of infection, interstitial lung disease, and bronchiectasis is similar in patients with PAD regardless of antibody deficiency type, but hematological malignancies are more common in patients with CVID, and connective tissue diseases are more common in patients with IgG-SGD [1].

In accordance with the literature, we first divided patients with PAD into two groups, CVID and IgG-SGD, based on clinical and immunological features, and then compared the groups. We then evaluated the patients by dividing them into four groups according to the clinical phenotypes they exhibited, regardless of antibody deficiency type. The distribution of clinical phenotypes in our patients was consistent with those of two different CVID cohorts we previously studied [22, 23]. According to our current study, respiratory diseases were the most common clinical phenotype, followed by gastroenterology/hepatology, rheumatology + dermatology, and hematology clinical phenotypes.

There were no differences in age or gender between patients with CVID and IgG-SGD. However, when patients were compared by clinical phenotype, the median age in the gastroenterology/hepatology and respiratory disease groups was lower than that in patients with other phenotypes. This result showed that patients in the gastroenterology/hepatology and respiratory diseases group exhibited the symptoms and signs earlier. In other words, there was a delay in diagnosis for patients in the hematology and rheumatology + dermatology groups. Although no studies in the literature have established a relationship between age at diagnosis and symptomatology, it has long been known that findings in the gastrointestinal and respiratory systems provide important clues for early diagnosis of PAD [24]. Although statistical comparisons could not be made between clinical phenotypes due to the small number of patients, the gender distribution was similar in the respiratory disease group, whereas the other clinical phenotypes showed a female predominance. In our previous study and some other published studies, it is noteworthy that the female gender is dominant in patients with PAD [1, 21, 23, 25, 26].

The IVIG doses administered to the patients were higher in the CVID group than in the IgG-SGD group. All of our patients diagnosed with IgG-SGD were receiving IVIG treatment because they were symptomatic, and a significant portion of them developed complications affecting different organs and systems. However, the administered IVIG dose was lower in patients with IgG-SGD than in those with CVID, consistent with the literature [27]. When patients were evaluated by clinical phenotype, no difference in IVIG dose was found between groups.

There was no significant difference in acute-phase reactants between the CVID and IgG-SGD patient groups, or between patient groups according to clinical phenotype. This was due to the patients being in a clinically and immunologically active or partially active period at the time of admission. Hemoglobin levels in peripheral blood, as well as white blood cell, neutrophil, lymphocyte, and platelet counts, were significantly lower in patients with CVID than in patients with IgG-SGD. When we compared patients by clinical phenotype, the same parameters were lower in the gastroenterology/hepatology and hematology groups than in other phenotypes, although the differences were not statistically significant. In biochemical tests, AST levels were higher in the gastroenterology/hepatology group than in the other groups. Total protein levels were lower in patients with CVID than in patients with IgG-SGD and lower in the gastroenterology/hepatology group than in other clinical phenotypes. These results were due to the clinical characteristics of the patient groups and were consistent with our previous findings [22, 23].

In our current study, significant differences were also observed in immunological tests. The levels of major immunoglobulin isotypes measured in the peripheral blood of patients with CVID were lower than those of patients with IgG-SGD. Additionally, the CD4/CD8 ratio in the first group was lower than that of the other group. In comparison, according to clinical phenotypes, IgG level, CD19^+^ B cell percentage, and the CD4/CD8 ratio were lower in our PAD patients with gastrointestinal system damage than in patients with other clinical phenotypes. In our previously published CVID cohorts, the CD4/CD8 ratio was low in patients with chronic diarrhea and low body weight, and the CD19^+^ B cell percentage and IgG levels were also low in these patients [22, 23]. Additionally, HLA-DR expression, indicative of chronic immunological activation, was elevated in CD3^+^ T cells from patients with a low CD4/CD8 ratio. When these studies are evaluated together, it is understood that clinical and immunological findings are further deteriorated in patients with PAD with gastrointestinal system involvement.

In our current study, FIB-4 scores were higher in patients with CVID than in those with IgG-SGD. Additionally, while there was a positive correlation between the FIB-4 score and acute-phase reactants (ESR, CRP) in patients with CVID, there was a negative correlation between this parameter and measurements of hemoglobin, leukocytes, neutrophils, albumin, and total protein. These results indicate that the FIB-4 score is associated with both acute and chronic inflammatory processes and may be important for the follow-up of patients with CVID to facilitate early diagnosis of liver damage. Indeed, we obtained similar findings when comparing patients with PAD by clinical phenotype. FIB-4 scores were higher in patients with gastroenterology/hepatology and hematology clinical phenotypes than in patients with other clinical phenotypes. Additionally, in the hematology group, the FIB-4 score showed a positive correlation with CRP and a negative correlation with IgG levels and CD19 percentage. These results indicate that as antibody levels decrease, the FIB-4 score increases, suggesting that the risk of liver damage may also increase. In our previous study, we found that AST and GGT levels were higher in CVID patients with hepatomegaly than in those without [22].

From a practical point of view, noninvasive, cost-effective screening tests are necessary to detect early stages of liver injury and fibrosis. The FIB-4 score is one of the most well-studied methods for liver fibrosis. Corey et al found a specificity of 88% and a positive predictive value of 76% for the FIB-4 score in detecting significant liver fibrosis in patients with NAFLD [28]. Harrison et al. reported that the FIB-4 score had good diagnostic accuracy for classifying fibrosis stage in patients with NASH, with a sensitivity of 64% and a specificity of 70% at a cutoff of 1.3 [29]. The recommended threshold values in guidelines for the FIB-4 score in stratifying liver fibrosis risk are: <1.3 as low risk, 1.3 to 2.67 as indeterminate risk, and >2.67 as high fibrosis risk [30]. Accordingly, considering the median FIB-4 scores of our patients with CVID and those with gastroenterology/hepatology and hematology clinical phenotypes, it is understood that they are in the indeterminate risk class for the development of liver fibrosis. As mentioned before, it can be easily predicted that patients in these groups are at higher risk for liver damage due to worse clinical and immunological findings. Indeed, the presence of immune thrombocytopenia in patients with CVID and those with a hematology clinical phenotype, and the detection of AST elevations in patients with a gastroenterology/hepatology clinical phenotype, directly affects the fibrosis risk score because the parameters in the FIB-4 formula are affected.

The strong and statistically significant positive correlation between the FIB-4 score and transient elastography in the gastroenterology/hepatology subgroup (*r* = 0.825, p < 0.001) demonstrates that these two noninvasive modalities provide highly consistent assessments of liver fibrosis. Specifically, the FIB-4 score’s ability to explain 64.5% of the variation in elastography values (R^2^ = 0.645) reflects a robust predictive power for identifying liver stiffness. Most importantly, our findings showed a 100% concordance rate (6/6 patients) between a high-risk FIB-4 score (>3.25) and advanced fibrosis stages (F3 or F4) on elastography. This perfect alignment in the high-risk category, coupled with a Cohen’s kappa coefficient of 0.588 (indicating moderate-to-good agreement), underscores that FIB-4 is not merely a marker of systemic inflammation but a reliable surrogate for architectural hepatic changes in PAD patients.

However, we also observed that FIB-4 is less definitive in the intermediate-risk category. The detection of significant fibrosis in some patients with scores <3.25 suggests that while FIB-4 is an excellent preliminary screening tool for identifying advanced disease, it lacks the sensitivity to entirely exclude significant fibrosis in low-to-moderate risk cases. Therefore, we recommend that patients in these categories undergo further evaluation with elastography or biopsy when clinical suspicion persists.

In our study, we also performed a multivariable analysis to identify independent predictors of elevated FIB-4 scores across the entire PAD cohort (n = 52). However, no significant independent associations were found between FIB-4 and the evaluated immunological or inflammatory parameters. This lack of uniform correlation across the broader population likely reflects the high clinical and immunological heterogeneity of PAD. These findings suggest that the clinical utility of the FIB-4 score may be phenotype-specific. The presence of documented liver damage at baseline in the gastroenterology/hepatology subgroup does not necessarily confound the results; rather, it provides a clinical validation of the FIB-4 score in the very population where noninvasive assessment of fibrosis is most urgently needed.

When we reviewed the literature, we did not find any other studies that examined whether the FIB-4 score is a risk factor for liver damage and fibrosis in PADs. The retrospective study conducted by Sterling et al. in patients with HIV/HCV coinfection causing secondary immunodeficiency was the first in this regard [11]. In this study, the authors suggested that the FIB-4 score is a useful noninvasive method for predicting liver fibrosis. In a similar study by Shah et al. in 237 patients with HIV/HCV coinfection, the FIB-4 score was compared with the AST to Platelet Ratio Index (APRI) score, and both noninvasive scoring systems were shown to be useful for identifying patients without advanced fibrosis with high accuracy [31].

Although this study’s results provide valuable clinical insights, it has certain limitations. The lack of correlation between FIB-4 scores and immunological parameters in CVID and PAD patients with a gastroenterology/hepatology phenotype was primarily due to the limited sample size and the nonnormal distribution of certain parameters. Consequently, the statistical power to detect subtle correlations within these specific subgroups was constrained. For the same reasons, stratifying patients into risk categories according to FIB-4 scores was not feasible. Furthermore, while differences in age and gender distribution were observed across clinical phenotypes, the small cohort size precluded a multivariable evaluation of how demographic characteristics influenced the measured parameters. Nonetheless, such sample-size constraints are inherent in clinical studies of rare PIDs, where large-scale cohorts are often difficult to recruit from a single center. Additionally, the subjective nature of patient histories limited the reliable evaluation of diagnostic delay, a known factor in the progression of chronic complications.

On the other hand, in the clinical follow-up of patients with PAD, noninvasive methods are prioritized to avoid the risks associated with invasive procedures (e.g., bleeding or infection in immunocompromised individuals). Accordingly, while FIB-4 was calculated for all patients, elastography was performed exclusively in the gastroenterology/hepatology subgroup based on clinical indications. Although this selective assessment may introduce selection bias and limit the generalizability of FIB-4–elastography comparisons to the entire PAD cohort, it reflects real-world clinical practice, where specialized imaging is prioritized for symptomatic patients. Consequently, the diagnostic performance of FIB-4 in asymptomatic PAD patients remains to be further validated in larger, prospective studies employing universal screening with elastography. Despite the correlation between FIB-4 and elastography, the absence of liver biopsy, the gold standard for fibrosis assessment, remains a significant limitation. While noninvasive tools are preferred in clinical practice for PAD patients due to their safety and accessibility, our findings regarding fibrosis severity should be interpreted with caution until further histopathological validation is available. Future prospective studies incorporating histopathological data, when ethically and clinically feasible, would be beneficial for further validating the diagnostic accuracy of the FIB-4 score in this specific population.

In conclusion, CVID and IgG-SGD, which are relatively common in the adult age group, develop chronic complications in the long term due to both late diagnosis and severe clinical course in some patients, and disease management becomes more difficult after this stage. Liver damage is one of the serious complications that increases morbidity and mortality in PAD. The FIB-4 score shows excellent agreement with elastography (positive predictive value), particularly in detecting significant fibrosis and cirrhosis. Therefore, this score is a cost-effective, noninvasive tool for early detection of significant liver damage in patients with PAD and should be closely monitored, especially in clinical phenotypes that affect platelet count and liver function tests. In patients with moderate FIB-4 risk, elastography should be used as a second-line test.

On the other hand, basic and advanced tests used in the diagnosis and follow-up of these diseases can be performed in almost every center where patients with PID are treated. An indirect result of this study is that clinical and immunological phenotypes can be determined in PADs using these tests, and that practical scoring systems can be developed in conjunction with biochemical parameters. However, to achieve more reliable results, subjective and objective data must be standardized and recorded from the moment the research is planned.

This study was approved by Baskent University Institutional Review Board and Ethics Committee (Project no: KA23/382). Written informed consent was received from all the participants. The study was conducted in accordance with the Declaration of Helsinki.

## Figures and Tables

**Table 1:** Comparisons of patients according to diagnosis of hypogammaglobulinemia and clinical phenotypes.

	Hypogammaglobulinemia			Clinical phenotypes						
	CVID n = 40 (76.9%)	IgG-SGD n = 12 (23.1%)	P ψ	H n = 7 (13.5%)	R&D n = 10 (19.2%)	G/H n = 14 (26.9%)	RED n = 21 (40.4%)	P ζ	P ψ	P ^ψ ψ^
**Age (years)**	45 ± 17	48 ± 15	0.606	60 (33–85) ^a^	63.5 (26–75) ^b^	44 (31–65) ^c^	38 (17–75) ^d^	**0.001**	^c^ vs. ^a^: .048, ^c^ vs. ^b^: .019, ^c^ vs. ^d^: .040	^a^ vs. ^d^: .018, ^b^ vs. ^d^: .001,
**Gender (F/M)**	30/10	9/3	0.635	6/1	10/0	12/2	11/10	NA	NA	-
**IVIG dose (g)**	39 ± 5.7	35 ± 3.6	**0.029**	40 (30–50)	35 (30–50)	40 (30–50)	40 (25–50)	0.051		
**ESR (mm/h)**	12 (2–32)	10 (2–31)	0.819	8 (4–27)	15.5 (2–32)	13 (2–32)	6 (2–31)	0.227		
**CRP**	7 (2–46)	6 (2–39)	0.390	7 (2–40)	9.6 (2–39)	7.9 (2–46)	6 (2–42)	0.243		
**Hgb (g/dL)**	13.2 (9.1–16.9)	14.4 (10.7–18.1)	**0.015**	13 (10.1–13.3) ^a^	13.9 (10.7–15.8) ^b^	13.2 (9.3–15.7) ^c^	14.2 (9.1–18.1) ^d^	**0.031**	^c^ vs. ^d^: .042	^a^ vs. ^b^: .040, ^a^ vs. ^d^: .021
**Leu (**/**mm^3^)**	5795 (1270–17,600)	9220 (5780–19,770)	**0.001**	4040 (1270–15,300) ^a^	9220 (6210–16,900) ^b^	4935 (1380–8090) ^c^	6450 (4170–19,770) ^d^	**0.001**	^c^ vs. ^b^ < .001, ^c^ vs. ^d^: .007	^a^ vs. ^b^: .032, ^b^ vs. ^d^: .035,
**Neu (**/**mm^3^)**	3480 (590–15,300)	5035 (2820–14,980)	**0.009**	2320 (690–5490) ^a^	5505 (3005–13,600) ^b^	3035 (590–5720) ^c^	4270 (2200–15,300) ^d^	**0.001**	^c^ vs. ^b^: .002, ^c^ vs. ^d^: .019	^a^ vs. ^b^: .005, ^a^ vs. ^d^: .014, ^b^ vs. ^d^: .035,
**Lym (**/**mm^3^)**	1655 (240–13400)	2450 (1320–4390)	**0.010**	1090 (240–13400) ^a^	2535 (1320–3270) ^b^	1365 (560–2170) ^c^	1960 (690–4390) ^d^	**0005**	^c^ vs. ^b^: .003, ^c^ vs. ^d^: .004	^-^
**Plt (10** **^3^** **/μL)**	180.2 (14–384)	302.5 (205–309)	**<0.001**	142 (30–212) ^a^	302.5 (166–399) ^b^	128.5 (14–247) ^c^	253 (18–361) ^d^	**<0.001**	^c^ vs. ^b^ < .001, ^c^ vs. ^d^ < .001	^a^ vs. ^b^: .001, ^a^ vs. ^d^ < .001, ^b^ vs. ^d^: .031
**ALT (IU)**	16.5 (9–65)	21.5 (8–42)	0.350	16 (9–42)	22.5 (11–42)	16.5 (11–49)	19 (8–65)	0.641	NA	NA
**AST (IU)**	26 (10–113)	24 (12–33)	0.192	30 (13–113) ^a^	21.5 (14–61) ^b^	36.5 (20–83) ^c^	20 (10–53) ^d^	**0.007**	^c^ vs. ^b^: .005, ^c^ vs. ^d^: .001	^-^
**Alb (g/dL)**	4.2 (2.7–5.5)	4.7 (3.2–5.5)	0.069	4.3 (2.8–5.1)	4.2 (3.2–5.1)	4.1 (2.9–4.8)	4.7 (2.7–5.5)	0.227	NA	NA
**T. prot (g/dL)**	5.9 (4.2–6.8)	7.5 (5.9–8.5)	**<0.001**	6.2 (5.1–6.5) ^a^	6.3 (5.9–8.2) ^b^	5.5 (4.2–6.3) ^c^	6.3 (5.2–8.5) ^d^	**0.001**	^c^ vs. ^a^: .036, ^c^ vs. ^b^ < .001, ^c^ vs. ^d^: .002	^-^
**FIB-4**	1.71 (0.31–20.47)	0.66 (0.36–1.39)	**0.003**	2.5 (1.09–20.08) ^a^	0.96 (0.36–1.95) ^b^	2.61 (1.43–20.47) ^c^	0.71 (0.31–1.47) ^d^	**<0.001**	^c^ vs. ^b^ < .001, ^c^ vs. ^d^ < .001	^a^ vs. ^b^: .002, ^a^ vs. ^d^ < .001
**Elastography (kPa)**	-	-	**-**	-	-	9.9 (5.1–32.5)	-	**-**	NA	NA
**IgG (g/L)**	4.1 (0.2–5.6)	6.2 (5.2–7.8)	**<0.001**	4.5 (2.8–5.5) ^a^	5.5 (4.1–7.8) ^b^	3.4 (0.2–4.7) ^c^	4.5 (2.6–7.6) ^d^	**<0.001**	^c^ vs. ^a^: .025, ^c^ vs. ^b^ < .001, ^c^ vs. ^d^: .007	^a^ vs. ^b^: .015, ^b^ vs. ^d^: .042,
**IgA (g/L)** *	15.0	83.3	**<0.001**	0.0	80.0	7.1	33.3	NA	NA	NA
**IgM (g/L)** *	15.0	66.7	**0.001**	42.9	40.0	0.0	33.3			
**IgE (IU/mL)** *	30.0	25.0	0.523	42.9	20.0	21.4	33.3			
**CD3 (%)**	79.1 (56.9–95.5)	74.2 (56–88.4)	0.165	72 (68.4–90.2) ^a^	76.3 (56–88.4) ^b^	86.9 (57.9–95.5) ^c^	78 (56.9–92.2) ^d^	**0.038**	^c^ vs. ^a^: .030, ^c^ vs. ^b^: .018, ^c^ vs. ^d^: .034	^-^
**CD4 (%)**	33.7 (13.7–56.4)	47.8 (23.6–65.3)	**0.002**	40.3 (24.9–55.6) ^a^	46.2 (19–65.3) ^b^	31.3 (13.7–56.4) ^c^	34 (15.3–52.4) ^d^	**0.038**	^c^ vs. ^b^: .030	^a^ vs. ^d^: .047, ^b^ vs. ^d^: .022
**CD8 (%)**	39.1 (13.8–67)	23.7 (12.0–36.4)	**<0.001**	29.7 (20.1–41.9) ^a^	19.3 (12–45) ^b^	48 (13.8–67) ^c^	35.9 (21.3–65) ^d^	**0.002**	^c^ vs. ^a^: .014, ^c^ vs. ^b^: .006,	^a^ vs. ^d^: .039, ^b^ vs. ^d^: .005
**CD4/CD8**	0.88 (0.21–3.11)	2.37 (0.85–3.82)	**<0.001**	1.43 (0.59–2.15) ^a^	2.95 (0.44–3.82) ^b^	0.73 (0.21–3.11) ^c^	0.96 (0.32–2.46) ^d^	**0.005**	^c^ vs. ^a^: .037, ^c^ vs. ^b^: .008	^a^ vs. ^d^: .021, ^b^ vs. ^d^: .008
**CD19 (%)**	9.4 (3.0–40.6)	10.8 (9–17.6)	0.368	14.2 (1.1–22) ^a^	11.2 (3–19) ^b^	4.1 (0–17.9) ^c^	8.9 (0.9–40.6) ^d^	**0.011**	^c^ vs. ^a^: .011, ^c^ vs. ^b^: .022, ^c^ vs. ^d^: .013	^-^
**CD3^−^CD16^+^56^+^ (%**)	10.8 (2–35)	14.1 (4.7–38)	0.580	10.8 (7.3–12) ^a^	11.2 (4.7–38) ^b^	8 (3.4–32.5) ^c^	9.9 (2–35.5) ^d^	0.580	NA	NA

Abbreviations: F: female, M: male, Hgb: hemoglobin, Leu: leukocyte, Neu: neutrophil, Lym: lymphocyte, Plt: platelet; ALT: alanine aminotransferase, AST: aspartate aminotransferase, Alb: albumin, T. prot: total protein; FIB-4: Index of liver fibrosis; kPa: kilopascal, IgG: Immunoglobulin G, CD: cluster of differentiation, H: hematology, R&D: rheumatology and dermatology, G/H: gastroenterology/hepatology, RED: respiratory diseases; NA: not applicable.

* Data were presented as the percentage of patients with a measurable level of the immunoglobulin isotype.

ζ p-values from Kruskal–Wallis test,

ψ p**-**values from Mann–Whitney *U* test.

The gastroenterology/hepatology phenotype and p ψ columns were highlighted in gray to indicate the primary role of this group in evaluating FIB-4 and liver-related parameters. Statistical comparisons given in the p ψ column focused on differentiating this high-risk subgroup from other clinical phenotypes to assess the clinical utility of the scoring system. Accordingly, pairwise comparisons between other PAD subgroups that do not involve the gastroenterology/hepatology phenotype were presented in the p ψ ψ column to maintain focus on the primary research objective and enhance clarity in the data presentation.

**Table 2:** Clinical and pathological spectrum of liver manifestations in the gastroenterology/hepatology subgroup ($n=14$).

Category of liver damage	Specific pathologies/comorbidities	No. of patients
Inflammatory bowel disease (IBD)	Ulcerative colitis, Crohn’s disease, or overlap	9
Vascular/infiltrative involvement	Nodular regenerative hyperplasia (NRH)	3
Autoimmune/chronic hepatitis	Type 1 autoimmune hepatitis, HBV/HCV carriage	3
Metabolic/steatotic damage	Grade 1 and grade 2 steatosis	5
Associated malabsorption	Celiac disease	1

**Table 3:** Contingency table for computing Cohen’s kappa coefficient (k).

	RATER 1			
RATER 2 RATER 2		Elastography: no significant fibrosis (<9.6 kPa)	Elastography: significant fibrosis (^3^9.6 kPa)	Total (FIB-4)
	FIB-4: no significant fibrosis (<3.25)	5 (a)	3 (b)	8
	FIB-4: significant fibrosis (^3^3.25)	0 (c)	6 (d)	6
	Total (elastography)	5	9	14

Compatibility (a + d): Number of patients for whom both tests give the same result: 5 + 6 = 11 patients. Incompatibility (b + c): Number of patients for whom the two tests gave different results: 3 + 0 = 3 patients.
